# Differential associations of *APOE-ε2* and *APOE-ε4* alleles with PET-measured amyloid-β and tau deposition in older individuals without dementia

**DOI:** 10.1007/s00259-021-05192-8

**Published:** 2021-02-01

**Authors:** Gemma Salvadó, Michel J. Grothe, Colin Groot, Alexis Moscoso, Michael Schöll, Juan Domingo Gispert, Rik Ossenkoppele, for the Alzheimer’s Disease Neuroimaging Initiative

**Affiliations:** 1 Alzheimer Prevention Program, Barcelonaβeta Brain Research Center (BBRC), Pasqual Maragall Foundation, C/ Wellington, 30 08005 Barcelona, Spain; 2 IMIM (Hospital del Mar Medical Research Institute), Barcelona, Spain; 3 Alzheimer Center Amsterdam, Department of Neurology, Amsterdam Neuroscience, Vrije Universiteit Amsterdam, Amsterdam UMC, Amsterdam, The Netherlands; 4 Wallenberg Centre for Molecular and Translational Medicine, Department of Psychiatry and Neurochemistry, Institute of Neuroscience and Physiology, University of Gothenburg, Gothenburg, Sweden; 5 Unidad de Trastornos del Movimiento, Servicio de Neurología y Neurofisiología Clínica, Instituto de Biomedicina de Sevilla (IBiS), Hospital Universitario Virgen del Rocío/CSIC/Universidad de Sevilla, Avda. Manuel Siurot, s/n 41013, Seville, Spain; 6 Dementia Research Centre, Institute of Neurology, University College London, London, UK; 7 Universitat Pompeu Fabra, Barcelona, Spain; 8 Centro de Investigación Biomédica en Red de Bioingeniería, Biomateriales y Nanomedicina (CIBER-BBN), Madrid, Spain; 9 Clinical Memory Research Unit, Lund University, Lund, Sweden

**Keywords:** Tau, Amyloid-β, Cross-sectional, Longitudinal, Sex interaction, Cognition, Hippocampal volumes, APOE, PET

## Abstract

**Purpose:**

To examine associations between the *APOE-ε2* and *APOE-ε4* alleles and core Alzheimer’s disease (AD) pathological hallmarks as measured by amyloid-β (Aβ) and tau PET in older individuals without dementia.

**Methods:**

We analyzed data from 462 ADNI participants without dementia who underwent Aβ ([^18^F]florbetapir or [^18^F]florbetaben) and tau ([^18^F]flortaucipir) PET, structural MRI, and cognitive testing. Employing *APOE-ε3* homozygotes as the reference group, associations between *APOE-ε2* and *APOE-ε4* carriership with global Aβ PET and regional tau PET measures (entorhinal cortex (ERC), inferior temporal cortex, and Braak-V/VI neocortical composite regions) were investigated using linear regression models. In a subset of 156 participants, we also investigated associations between *APOE* genotype and regional tau accumulation over time using linear mixed models. Finally, we assessed whether Aβ mediated the cross-sectional and longitudinal associations between *APOE* genotype and tau.

**Results:**

Compared to *APOE-ε3* homozygotes, *APOE-ε2* carriers had lower global Aβ burden (β_std_ [95% confidence interval (CI)]: − 0.31 [− 0.45, − 0.16], *p* = 0.034) but did not differ on regional tau burden or tau accumulation over time*. APOE-ε4* participants showed higher Aβ (β_std_ [95%CI]: 0.64 [0.42, 0.82], *p* < 0.001) and tau burden (β_std_ range: 0.27-0.51, all *p* < 0.006). In mediation analyses, *APOE-ε4* only retained an Aβ-independent effect on tau in the ERC. *APOE-ε4* showed a trend towards increased tau accumulation over time in Braak-V/VI compared to *APOE-ε3* homozygotes (β_std_ [95%CI]: 0.10 [− 0.02, 0.18], *p* = 0.11), and this association was fully mediated by baseline Aβ.

**Conclusion:**

Our data suggest that the established protective effect of the *APOE-ε2* allele against developing clinical AD is primarily linked to resistance against Aβ deposition rather than tau pathology.

**Supplementary Information:**

The online version contains supplementary material available at 10.1007/s00259-021-05192-8.

## Introduction

The apolipoprotein-E (*APOE*) *ε4* allele is the major genetic risk factor for sporadic Alzheimer’s disease (AD). *APOE-ε4* is associated with increased levels of amyloid-β (Aβ) [1–4] and tau aggregates [5–9], the two main pathological hallmarks of AD. However, the question whether *APOE-ε4* directly impacts tau pathology or increases tau in an Aβ-dependent fashion remains controversial [10–14].

Contrary to the detrimental effect of *APOE-ε4*, the *APOE-ε2* allele is protective against AD [2, 15, 16]. Studies focusing on *APOE*-*ε2* are scarce due to the low prevalence of this allele in the general population (~ 8%) and especially in AD populations (~ 5%) [17, 18]. Previous cerebrospinal fluid (CSF) and PET studies have reported robust associations between *APOE*-*ε2* and decreased levels of Aβ pathology [19–21]. Regarding tau pathology, nearly all biomarker studies on this topic reported no association with the *APOE*-*ε2* allele [19, 21, 22], with one exception reporting lower phosphorylated tau (p-tau) [23]. However, all these studies measured the amount of tau pathology in CSF, which provides information on global soluble p-tau burden but not on the regional distribution of neurofibrillary tau tangles as provided by tau-sensitive PET imaging techniques. Of note, multiple studies have assessed the concordance between CSF tau and tau PET reporting a moderate association [24–26]; however, this association may be dependent on disease stage, neurodegeneration, and the tau fragments measured in CSF [27, 28]. Furthermore, a recent study showed that dichotomization of PET and CSF-derived tau measures could lead to ~ 25% discordance, which does impact future cognitive decline [29]. Thus, potential associations between *APOE*-*ε2* and regional tau deposition on PET remain to be investigated, which is all the more important as a recent PET study has identified a clear regional predilection for *APOE-ε4*-related tau deposition confined to the medial temporal lobe [9]. Given that this association was found to be partly independent of amyloid-β aggregation, *APOE* was even proposed as a potential target for anti-tau disease-modifying therapies.

In the present study, we leveraged available *APOE* genotyping and multitracer PET imaging data from a large cohort of older individuals without dementia to study cross-sectional and longitudinal associations of the *APOE-ε2* and *ε4* alleles with regional tau deposition and put these in the context of *APOE* effects on Aβ pathology. At a cross-sectional level, we hypothesized that *APOE-ε4* participants would have higher levels of both Aβ and tau pathology as previously reported, and that the *APOE-ε2* allele would be associated with lower Aβ—but not tau—burden. We further hypothesized that Aβ would mediate the effect of *APOE-ε4* on tau differently depending on the brain region under investigation, as a previous PET study reported an Aβ-independent association with tau pathology in the medial temporal lobe, but not in the rest of the brain [9]. In the longitudinal analysis, we hypothesized to observe faster increase in tau accumulation in *ε4* carriers, but no difference in accumulation rates in *ε2* carriers compared to *APOE-ε3* homozygotes. Finally, as recent studies have indicated a possible interaction effect between the *APOE*-*ε4* allele and female sex on both Aβ and tau pathology [4, 8, 21, 30, 31], we also investigated this in the current PET study. Our study, assessing the impact of genetic variants on Aβ and tau imaging, will contribute evidence required for their diagnostic use, as outlined in the strategic biomarker roadmap for the validation of AD diagnostic biomarkers [32].

## Material and methods

### Participants

From the Alzheimer’s Disease Neuroimaging Initiative (ADNI) database, we selected all participants without dementia (i.e., cognitively unimpaired [CU] or diagnosed with mild cognitive impairment [MCI]) that underwent Aβ PET ([^18^F]florbetapir or [^18^F]florbetaben), tau PET ([^18^F]flortaucipir), and magnetic resonance imaging (MRI) (*n* = 462). Participants were grouped as *APOE-ε2* carriers (i.e., 1 or 2 *APOE-ε2* alleles; *n* = 45), *APOE-ε3* homozygotes (*n* = 257), or *APOE-ε4* carriers (i.e., 1 or 2 *APOE-ε4* alleles, *n* = 160). We excluded *APOE-ε2ε4* participants (*n* = 7) from further analyses. A subset of 156 individuals (10 *APOE-ε2*, 76 *APOE-ε3ε3*, 70 *APOE-ε4*) also underwent at least one follow-up [^18^F]flortaucipir tau PET scan on average 1.6 (0.7) years later.

We included CU and MCI patients because we aimed to investigate how *APOE* modified the early development of Aβ and tau pathology, and *APOE* is a known AD-risk factor in these individuals [1]. On the other hand, it has previously been reported that patients with AD dementia showed different associations between *APOE* and Aβ and tau biomarkers, compared to patients without dementia [33–35]. Thus, to avoid possible bias in our results, we excluded AD dementia patients from our analyses. Although *APOE* effects may be influenced by diagnosis status (i.e., CU or MCI), we pooled these two groups to increase the sample size (especially for *ε2* carriers, the main *APOE* allele of interest in our study) while adjusting for clinical diagnosis in the statistical models (please see the “Statistical analyses” section).

ADNI is a multi-site open access dataset designed to accelerate the discovery of biomarkers to identify and track AD pathology (adni.loni.usc.edu/). Data collection and sharing in ADNI were approved by the Institutional Review Board of each participating institution, and written informed consent was obtained from all participants.

### *APOE* genotyping

*APOE* genotype was determined by genotyping the two single-nucleotide polymorphisms that define the *APOE-ε2*, *ε3*, and *ε4* alleles (rs429358, rs7412) with DNA extracted by Cogenics from a 3-mL aliquot of EDTA blood (adni.loni.usc.edu/data-samples/ genetic-data/).

### Image acquisition

All image acquisition procedures are described in detail on the ADNI website (http://adni.loni.usc.edu/methods/documents/). Briefly, [^18^F]flortaucipir tau PET images were acquired in six frames of 5 min each, 75-105-min post-injection (p.i.). Aβ PET images were acquired in four frames of 5 m each, 50-70 min p.i. for [^18^F]florbetapir and 90-110 min p.i. for [^18^F]florbetaben. Finally, structural MRI data were acquired on 3-T scanning platforms using T1-weighted sagittal 3-dimensional magnetization-prepared rapid-acquisition gradient echo sequences (MP-RAGE).

### Image processing

Regional tau PET data were downloaded from the ADNI Laboratory of Neuroimaging (LONI) database (https://ida.loni.usc.edu). The full preprocessing pipeline is specified elsewhere [36]. In brief, first the T1-weighted MR image closest in time to the [^18^F]flortaucipir PET scan was segmented in native space using Freesurfer (v5.3.0). Then, each [^18^F]flortaucipir image was co-registered to the MRI. Finally, the MR-segmented regions were used to calculate mean volume-weighted uptake in the corresponding region on PET. Standardized uptake value ratios (SUVR) were computed using inferior cerebellar gray matter as reference region. For this study, we selected the following regions-of-interest (ROI): the entorhinal cortex (ERC), inferior temporal cortex (ITC), and a neocortical Braak V/VI composite region [37], representing early, intermediate, and late regions of tau accumulation, respectively. The hippocampus was not merged with the ERC into an early Braak I/II region due to known [^18^F]flortaucipir PET signal confounds in this region [38, 39].

For Aβ PET, we downloaded global neocortical composite SUVR values (using the whole cerebellum as reference) from the ADNI-LONI database (adni.loni.usc.edu/methods/pet-analysis). We converted these values to the common Centiloid scale using equations previously derived by the ADNI PET Core (http://adni.loni.usc.edu/data-samples/access-data/) in order to be able to combine data from [^18^F]florbetapir and [^18^F]florbetaben scans [40].

Preprocessed structural MR images were downloaded from the ADNI server to calculate hippocampal volumes. We extracted the hippocampal gray matter volume using an automated atlas-based volumetry approach based on a hippocampal standard space mask representing harmonized delineation criteria as described previously [41]. Total intracranial volumes, calculated as the total sum of segmented gray matter, white matter, and CSF volumes, were used to normalize the hippocampal volumes.

### Neuropsychological assessment

We downloaded the widely used composite scores of episodic memory (ADNI-MEM) [42] and executive function (ADNI-EF) [43] from the ADNI-LONI website, as well as the recently validated composites of language (ADNI-LAN) and visuospatial functioning (ADNI-VS) [44].

### Statistical analyses

We compared demographic information between *APOE* groups in the main sample and the longitudinal subsample using ANOVA for continuous variables and Chi-squared tests for categorical variables.

The primary cross-sectional analysis assessed the associations between *APOE* alleles and the two main hallmarks of AD (Aβ and tau) measured with PET. We performed this analysis using linear regression with *APOE* groups (i.e., *ε2* carriers, *ε3* homozygotes, and *ε4* carriers) as independent variable, and Centiloids (global Aβ PET measure), ERC, ITC, and Braak V/VI [^18^F]flortaucipir SUVR (regional tau PET measures) as dependent variables, while adjusting for age, sex, education, and diagnosis (CU or MCI), in independent models. Gene-dose effects were not investigated due to the complete lack of *ε2* homozygotes in the sample. We also examined *APOE* by sex interaction effects on Aβ and tau PET variables. As a secondary analysis, we examined *APOE* by Aβ status interactions on tau PET burden. To that end, we dichotomized Aβ load using a previously validated threshold of > 12 Centiloids, which has been shown to sensitively detect early Aβ pathology in an independent neuropathologic validation study and a CSF study [45, 46]. Additionally, we investigated main *APOE* effects on normalized hippocampal volumes and cognitive composite scores using analog covariate-controlled linear regression models.

We performed *post hoc* mediation analyses to test whether the associations between *APOE* and regional tau PET burden were mediated by global Aβ burden. Age, sex, education, and diagnosis were included as covariates in these models and this analysis was only performed for associations that were significant in the primary analysis described above. We repeated this mediation analysis when stratifying by sex and Aβ status.

In a complementary longitudinal analysis on a subset of participants with serial tau PET acquisitions (*n* = 156), we assessed whether the *APOE* groups showed differential rates of tau accumulation over time. To this end, we applied linear mixed models with random slopes and intercepts. Regional tau burden (ERC, ITC, and Braak V/VI) served as dependent variables, and we included main effects for time and *APOE* groups as well as an interaction term for time*APOE. These models were adjusted for age, sex, education, and diagnosis. Note that we performed this analysis only for tau PET and not Aβ PET, because longitudinal Aβ PET imaging was performed before tau PET and our primary focus was on *APOE* associations with tau PET.

To examine whether longitudinal tau accumulation was dependent on Aβ burden at baseline, we performed another mediation analysis using the rate of tau accumulation as dependent variable and baseline Aβ load as mediator. Rates of tau accumulation were calculated by extracting the subject-specific slopes from a linear mixed model including only time as predictor. Age, sex, education, and diagnosis at baseline were included as covariates in all paths of the mediation analysis. Mediation analyses were only performed on the associations that were significant or showed a trend towards significance in the previous analysis.

For all analyses, *APOE-ε3ε3* participants were selected as the reference group. Statistical significance was set at *p* < 0.05. Following recommendations described in the statistical literature, we did not apply a correction for multiple comparisons in this hypothesis-driven study with a limited number of planned comparisons that are motivated by previous literature [47] as the main analysis. However, we included multiple comparisons-adjusted results of the main analyses as supplementary information. We used the *mvt* method of the *emmeans* package to perform these adjustments. All statistical analyses were performed with R (v3.6.2), except mediation analyses that were performed with the PROCESS (v3.4.1) toolbox from SPSS (www.processmacro.org) [48]. Statistics were derived using a bootstrapping approach with *n* = 1000 iterations as implemented in the R package *boot*.

## Results

### Sample characteristics

Participant demographics, imaging, and cognitive data of the study sample are presented in Table 1. Participants were mainly CU (67.9%), had a mean age of 74.3 years and evenly distributed sex (52.8% women). Across *APOE* groups, *ε4* carriers were slightly younger than ε2 carriers and *ε3* homozygotes (*F* = 3.940, *p* = 0.020). There were no other differences in demographic characteristics across *APOE* groups. Out of the 462 participants, 324 underwent [^18^F]florbetapir Aβ PET (70.1%) and 138 [^18^F]florbetaben Aβ PET (29.9%). *APOE*-*ε4* carriers had a higher proportion of Aβ-positive subjects (74.4%, *p* < 0.001) and *APOE-ε2* carriers a lower proportion (33.3%, *p* = 0.054) compared to the *ε3* homozygotes (49.0%). Characteristics of the subsample with longitudinal tau PET did not differ significantly from the main sample on any of the variables (Table s1). Demographic information stratified by *APOE* and sex and by *APOE* and Aβ status are shown in Table s2 and Table s3, respectively.

**Table 1:** Demographics, imaging, and cognitive information of the study sample. Values reflect group means and standard deviations in parentheses, unless otherwise specified. Aβ status was positive (negative) if Aβ load was higher (lower) than 12 Centiloids [45, 46]. *p*-values correspond to the overall group (i.e., *APOE* genotype) effects, while superscript letters indicate significance of the pair-wise group differences (a: *APOE-ε4* carriers vs. *APOE-ε3ε3*; b: *APOE-ε2* carriers vs. *APOE-ε4* carriers)

	Main sample				
	All (*n* = 462)	*APOE-ε2* carriers (*n* = 45)	*APOE-ε3ε3* (*n* = 257)	*APOE-ε4* carriers (*n* = 160)	*p*
Demographics					
Age, years	74.3 (7.6) [56-94]	74.7 (6.8) [61-92]	75.1 (7.8) [56-94]	72.9 (7.3) [57-94]	**0.020** ^a^
Women, *n* (%)	244 (52.8)	21 (46.7)	132 (51.4)	91 (56.9)	0.376
Education, years	16.6 (2.5)	16.3 (2.6)	16.8 (2.4)	16.4 (2.6)	0.282
Diagnosis, CU *n* (%)	315 (68.2)	29 (64.4)	184 (71.6)	102(63.8)	0.210
MMSE	28.4 (2.5)	28.6 (1.8)	28.4 (2.6)	28.2 (2.4)	0.443
Tau PET measurements (SUVR)					
ERC	1.19 (0.20)	1.16 (0.16)	1.15 (0.17)	1.26 (0.24)	**< 0.001**^a,b^
ITC	1.26 (0.24)	1.22 (0.10)	1.24 (0.22)	1.32 (0.29)	**0.002** ^a,b^
Braak V/VI	1.09 (0.14)	1.06 (0.07)	1.08 (0.13)	1.12 (0.15)	**0.005** ^a,b^
Aβ PET measurement					
Centiloids	32.1 (37.1)	14.4 (14.8)	25.5 (33.6)	47.6 (41.4)	**< 0.001**^a,b^
Aβ positive, *n* (%)	260 (56.3)	15 (33.3)	126 (49.0)	119 (74.4)	**< 0.001**^a,b^
MRI measurement					
Hippocampal volumes	4.56 (0.52)	4.53 (0.51)	4.57 (0.51)	4.57 (0.54)	0.903
Cognitive composite measures (*z* scores)					
Episodic memory	0.78 (0.73)	0.73 (0.71)	0.83 (0.71)	0.71 (0.77)	0.262
Executive function	0.86 (0.93)	0.82 (0.71)	0.90 (0.97)	0.83 (0.91)	0.685
Language	0.68 (0.85)	0.64 (0.77)	0.67 (0.87)	0.69 (0.85)	0.943
Visuospatial functioning	0.12 (0.69)	0.22 (0.67)	0.12 (0.70)	0.10 (0.67)	0.596

Significant *p*-values (*p* < 0.05) are shown in bold.

*CU* cognitively unimpaired; *MMSE* Mini-Mental State Examination; *SUVR* standardized uptake value ratio; *ERC* entorhinal cortex; *ITC* inferior temporal cortex; *Aβ* amyloid-β; *MRI* magnetic resonance imaging

### Associations between *APOE* alleles and cross-sectional Aβ and tau PET

**Fig. 1:**
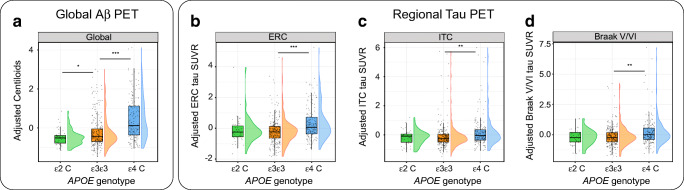
Associations of *APOE-ε2* and *APOE-ε4* alleles with cross-sectional measures of Aβ (**a**) and tau burden (**b**-**d**). Tau regions studied were ERC (**b**), ITC (**c**), and Braak V-VI (**d**). PET measures are adjusted by age, sex, education, and diagnosis. Boxplots show median values (middle line) with lower and upper hinges corresponding to the first and third quartiles. Dots represent individual adjusted PET measures, with violin plots showing their distribution. Aβ, amyloid-β; ERC, entorhinal cortex; ITC, inferior temporal cortex; SUVR, standardized uptake value ratio. * *p* < 0.05; *** *p* < 0.001

Compared to *APOE-ε3ε3* participants, *ε2* carriers showed lower global Aβ burden (β_std_ [95% confidence interval (CI)] = − 0.31 [− 0.45, − 0.16], *p* = 0.034), but no differences in regional tau burden (Table 2 and Fig. 1). On the contrary, *ε4* carriers exhibited higher Aβ load (β_std_ [95%CI]: 0.64 [0.42, 0.82], *p* < 0.001) as well as higher tau load in all regions assessed (ERC: β_std_ [95%CI] = 0.51 [0.33, 0.70], *p* < 0.001; ITC: β_std_ [95%CI] = 0.30 [0.08, 0.49], *p* = 0.002; Braak V/VI: β_std_ [95%CI] = 0.27 [0.03, 0.49], *p* = 0.006; Table 2 and Fig. 1). Results corrected for multiple comparisons can be found in Table s4.

**Table 2:** Linear regression parameters (standardized β) for the association of *APOE-ε2* and *APOE-ε4* genotypes with tau PET, Aβ PET, MRI, and cognition measurements. *APOE-ε3ε3* participants were selected as the reference group for all comparisons*.* Models included age, sex, education, and diagnosis as covariates

	*APOE-ε2*		*APOE-ε4*	
	β [95%CI]	*p*	β [95%CI]	*p*
Tau measurements				
ERC	0.03 [− 0.17, 0.39]	0.533	**0.51 [0.33, 0.70]**	**< 0.001**
ITC	− 0.10 [− 0.26, 0.10]	0.498	**0.30 [0.08, 0.49]**	**0.002**
Braak V-VI	− 0.12 [− 0.32, 0.08]	0.449	**0.27 [0.03, 0.49]**	**0.006**
Aβ measurement				
Centiloids	− **0.31 [**− **0.45,** − **0.16]**	**0.034**	**0.64 [0.42-0.82]**	**< 0.001**
MRI measurement				
Hippocampal volumes	− 0.02 [− 0.35, 0.26]	0.461	− 0.08 [− 0.24, 0.10]	0.331
Cognitive composite measures				
Episodic memory	− 0.05 [− 0.30, 0.24]	0.475	− **0.17 [**− **0.33, 0.01]**	**0.048**
Executive function	− 0.04 [− 0.27, 0.23]	0.519	− 0.10 [− 0.27, 0.07]	0.241
Language	0.05 [− 0.25, 0.32]	0.451	0.03 [− 0.14, 0.22]	0.468
Visuospatial functioning	0.18 [− 0.12, 0.48]	0.246	− 0.03 [− 0.22, 0.18]	0.474

Statistically significant results (*p* < 0.05)

*Aβ* amyloid-β; *MRI* magnetic resonance imaging; *CU* cognitively unimpaired; *CI* confidence interval

There was also a sex*APOE-ε4 interaction effect on ERC tau burden (β_stand_ [95%CI]: 0.39 [0.03, 0.76], *p* = 0.038; Table 3), with the deleterious effects of the *ε4* allele on tau being higher in women. Detailed results of the sex-stratified analyses are provided in Table s5. The significant interaction between Aβ status and *APOE-ε4* on ERC tau burden (β_stand_ [95%CI]: 0.62 [0.28, 0.91], *p* = 0.002; Table 3) indicates a stronger *APOE-ε4* effect on tau burden in Aβ-positive participants. Detailed results of the analyses stratified by Aβ status are provided in Table s6 and Figure s1. Results of the sex and Aβ status interactions corrected for multiple comparisons can be found in Table s7.

**Table 3:** Linear regression parameters (standardized β) for the interaction between *APOE* and sex, and *APOE* and Aβ status on tau and Aβ load. *APOE-ε3ε3* men and *APOE-ε3ε3* Aβ negative were selected as the reference group for sex and Aβ status comparisons, respectively*.* Aβ status was positive (negative) if Aβ load was higher (lower) than 12 Centiloids [45, 46]. All models included age, education, and diagnosis as covariates

	APOE-ε2*sex		APOE-ε4*sex		APOE-ε2*Aβ status		APOE-ε4*Aβ status	
	β [95%CI]	*p*	β [95%CI]	*p*	β [95%CI]	*p*	β [95%CI]	*p*
Tau measurements								
ERC	− 0.18 [− 0.82, 0.24]	0.469	**0.39 [0.03-0.76]**	**0.038**	− 0.35 [− 0.88, 0.07]	0.256	**0.62 [0.28, 0.91]**	**0.002**
ITC	0.04 [− 0.33, 0.40]	0.679	0.12 [− 0.26, 0.56]	0.402	− 0.13 [− 0.55, 0.20]	0.629	0.38 [− 0.03, 0.67]	0.067
Braak V/VI	0.13 [− 0.27, 0.59]	0.609	0.12 [− 0.29, 0.54]	0.403	− 0.07 [− 0.50, 0.39]	0.645	0.30 [− 0.13, 0.62]	0.161
Aβ measurement								
Centiloids	0.08 [− 0.23, 0.39]	0.676	0.15 [− 0.27, 0.48]	0.342	-	-	-	-

Statistically significant results (*p* < 0.05) are shown in bold

*Aβ* amyloid-β; *MRI* magnetic resonance imaging; *CU* cognitively unimpaired; *CI* confidence interval

Regarding non-PET measures, neither *APOE*-*ε2* nor *APOE*-*ε4* were significantly associated with hippocampal volumes. *APOE-ε4* carriers showed lower memory scores (β_stand_ [95%CI]: − 0.17 [− 0.33, 0.01], *p* = 0.048), but no other associations were observed between *APOE* and non-amnestic cognitive domain scores (*p* > 0.24, Table 2).

**Fig. 2:**
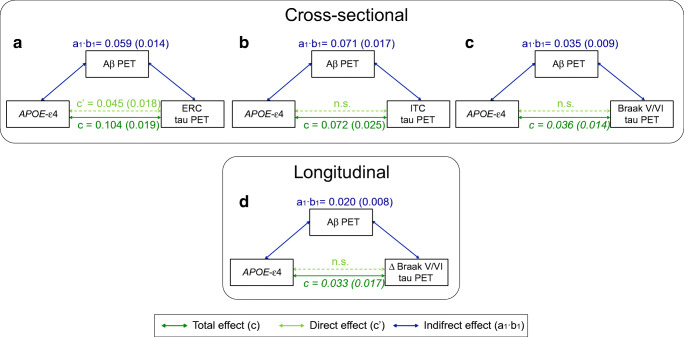
Mediation effect of Aβ on the association of *APOE-ε4* with cross-sectional (top) and longitudinal (bottom) tau deposition in the ERC (**a**), ITC (**b**), and Braak V-VI (**c** and **d**). Dark green lines show the total effect of *APOE-ε4* allele on tau burden, light green lines show the direct effect (i.e., without mediation), and blue lines depict the Aβ mediation effect. Path weights are only shown for significant paths and are displayed as (unstandardized) beta values with standard errors in brackets. Significance of the indirect effect was determined using bootstrapping with 5000 iterations. All models were adjusted by age, sex, education, and diagnosis. Aβ, amyloid-β; ERC, entorhinal cortex; ITC, inferior temporal cortex

In the mediation analyses, we found that the association between *APOE-ε4* and tau burden was mediated by global Aβ burden (Fig. 2, Table 4), but this relationship was not homogeneous across brain regions. Specifically, in the ERC, Aβ only partially mediated the association between *APOE-ε4* and tau burden, leaving a significant Aβ-independent effect of *APOE-*ε4 on tau burden that corresponded to 43% of the total effect (Table 4). On the other hand, Aβ fully mediated the association between *APOE-ε4* and tau in the ITC and Braak V/VI regions, indicating that there was no Aβ-independent effect of *ε4* on tau burden in these regions. Sex-stratified analyses revealed that the Aβ-independent *APOE-ε4* effect on tau burden in the ERC was only significant in women (Table s8). Restricting the mediation analyses to the Aβ-positive group revealed similar results as the main analyses. There was a significant Aβ-independent effect on ERC tau, whereas the *APOE-ε4* association with tau levels in the ITC was fully mediated by Aβ load (Table s9, Figure s2).

**Table 4:** Parameters of the mediation analyses. In the mediation analyses, the dependent variable (*X*) is the *APOE-ε4* allele, the mediator (*M*) is the baseline Aβ burden, measured as Centiloids, and the dependent variable (*Y*) is either the baseline tau PET SUVR (cross-sectional) or rate of tau PET SUVR change over time (longitudinal) in the different ROIs. The first three columns of each analysis show path weights (SE), while the last two columns show the percentage over the total effect. Mediation analyses were only performed for *APOE* associations with tau PET measures that were significant or showed a trend towards significance in the main analysis

	Total effect (*c*)	Mediated effect (*a*_1_·*b*_1_)	Direct effect (*c*′)	Percentage mediation (*a*_1_·*b*_1_/*c*)	Percentage direct (*c*′/*c*)
Cross-sectional					
ERC	0.104 (0.019)	0.059 (0.014)	0.045 (0.018)	56.7%	43.3%
ITC	0.072 (0.025)	0.071 (0.017)	n.s.	98.6%	n.s.
Braak V/VI	*0.036 (0.014)*	0.035 (0.009)	n.s.	97.2%	n.s.
Longitudinal					
Δ Braak V/VI	*0.033 (0.017)*	0.020 (0.008)	n.s.	60.6%	n.s.

Only paths that were statistically significant (*p* < 0.05) or showed a trend to significance (*p* < 0.1, in italics) are shown

*ERC* entorhinal cortex; *ITC* inferior temporal cortex; *n.s.* not significant; *Aβ* amyloid-β; *ROI* region of interest; *SE* standard error

### *APOE* associations with tau accumulation over time

**Fig. 3:**
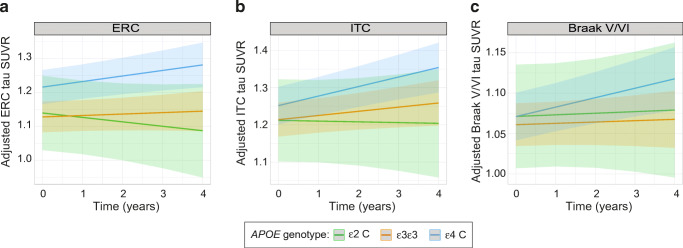
Associations of *APOE-ε2* and *APOE-ε4* alleles with longitudinal measures of regional tau accumulation. Linear slopes of tau PET SUVR change over time and their 95% confidence intervals, as determined from linear mixed models, are depicted for the different *APOE* genotypes and for the following regions: ERC (**a**), ITC (**b**), and Braak V-VI (**c**). PET measures are adjusted by age, sex, education, and diagnosis. ERC, entorhinal cortex; ITC, inferior temporal cortex. * *p* < 0.05

In the subsample with longitudinal tau PET data, none of the regional tau accumulation rates was significantly different between *ε4* carriers and *ε3* homozygotes, although *ε4* carriers showed a trend towards a steeper increase in tau burden in Braak V/VI regions (β_std_ [95%CI]: 0.10 [− 0.02, 0.18], *p* = 0.111) (Fig. 3, Table 5). *APOE-ε2* carriers did not show any differences in regional tau accumulation rates compared to *APOE-ε3ε3* participants (Fig. 3, Table 5). Multiple comparisons-corrected results are detailed in Table s10.

**Table 5:** Linear mixed model regression parameters (standardized β) for the association of *APOE-ε2* and *APOE-ε4* genotypes with longitudinal rates of regional tau SUVR change over time. *APOE-ε3ε3* participants were selected as the reference group for all comparisons. The model included age at baseline, sex, education, and diagnosis as covariates

	APOE-ε2*time		APOE-ε4*time	
	β [95%CI]	*p*	β [95%CI]	*p*
ERC	− 0.10 [− 0.25, 0.07]	0.396	0.08 [− 0.04, 0.15]	0.200
ITC	− 0.07 [− 0.17, 0.07]	0.505	0.08 [− 0.01, 0.15]	0.147
Braak V/VI	− 0.01 [− 0.18, 0.18]	0.629	0.10 [− 0.02 to 0.18]	0.111

*Aβ* amyloid-β; *ERC* entorhinal cortex; *ITC* inferior temporal cortex; *CI* confidence intervals

In mediation analyses, the association between *APOE-ε4* and increased rate of tau accumulation in Braak V/VI was fully mediated by baseline Aβ load (Table 4, Fig. 3). This effect remained significant when using baseline tau load as an additional covariate.

## Discussion

In this PET study, we investigated the relationship of *APOE-ε2* and *APOE-ε4* alleles with Aβ and tau load, the two main pathological hallmarks of AD in older individuals without dementia. We found an association of the *ε2* allele with reduced Aβ load but not with tau burden. Furthermore, we found that *ε4* carriers showed higher load of both Aβ and tau, and also a trend towards higher rate of longitudinal tau accumulation in neocortical regions representing advanced Braak tau stages V/VI. However, it is important to note that all associations between the *ε4* allele and baseline tau levels as well as rates of tau accumulation were at least partially mediated by Aβ. Furthermore, only the earliest tau deposition region (ERC) showed an Aβ-independent association between the *ε4* allele and tau load. Taken together, our data suggest that the protective effect of the *APOE-ε2* allele for developing clinical AD appears to be primarily linked to resistance against Aβ deposition [49] rather than tau pathology, and that the effect of the *APOE-ε4* allele on tau burden is mostly secondary to the prominent effect on increased Aβ load.

The finding of an association between *ε2* carriership and Aβ, but not with tau, is in accordance with previous studies assessing Aβ and p-tau levels in CSF [19, 21, 22]. The novelty of our study is the use of tau PET imaging, which allowed us to rule out the possibility of potential *APOE-ε2* effects on regional neurofibrillary tau burden not captured by CSF-based tau biomarkers (i.e., providing a single value representing the entire brain). In addition, PET-based and CSF-based biomarkers appear to reflect different aspects of tau pathology that may also be differentially affected by *APOE* genotype. In this context, it has been recently shown that tau PET is more closely related to cognition and atrophy than tau levels in CSF [28]. On the other hand, tau abnormalities might be detected earlier in CSF than on PET [50]. However, in addition to the missing *APOE-ε2* effect on regional tau burden in the cross-sectional PET data, here we also found that tau accumulation over time in serial PET acquisitions was not significantly attenuated in *APOE-ε2* carriers compared to *APOE-ε3* homozygotes. Before the advent of tau PET tracers, only neuropathological studies could investigate *APOE* effects on regional tau pathology. However, the association between the *APOE-ε2* allele and tau deposition in post-mortem studies has been controversial, as some studies showed no association [51, 52], while others reported lower levels of tau in *APOE-ε2* carriers [6, 10, 53, 54]. Difference in methodology across studies is a likely explanation for this disparity. For example, some of the older studies only found differences when comparing *ε2* carriers to non-carrier groups that also included *ε4* carriers, or included very low numbers of *ε2* carriers (*n* < 5) [6, 54]. In another study, *ε2* allele associations with tau were only found in the hippocampus, whereas this association was not significant in the neocortex nor in the ERC [53]. Finally, *APOE* effects on regional tau burden may be affected by differences in disease stage across *APOE* groups, where *ε2* carriers are typically also less clinically advanced than *ε3* homozygotes, which may explain their lower levels of tau pathology [10]. In the current study, we only included participants with no or only mild cognitive impairment and controlled all analyses for cognitive status.

As secondary outcomes, we investigated associations of the *ε2* allele with hippocampal volumes and cognition, but we did not find any statistically significant associations. This is in agreement with a previous multimodal study examining differences between *ε2* carriers and *ε3* homozygotes, in which these groups only differed in terms of Aβ burden but not on hippocampal volumes or memory performance (tested cross-sectionally and longitudinally) [19]. We expand on these findings by also including executive function measures and the new ADNI composite measures for language and visuospatial functioning, which have been shown to predict diagnostic conversion from MCI to AD dementia and are associated with MRI and CSF measurements [44]. While a positive effect of *APOE-ε2* carriership on preserved cognitive function in advanced age would be expected based on its known protective effect for developing AD dementia, the sample size of our PET-based sample may not provide sufficient power to detect such an effect [55, 56].

In contrast to our findings in *ε2* carriers, *ε4* carriers showed higher levels of both Aβ and tau in all the regions we investigated. Of note, we found that the effect size of the association between *ε4* and tau was higher in regions of early tau deposition (ERC > ITC > Braak V/VI). Results related to Aβ are in agreement with many previous studies showing higher levels of this AD pathological hallmark in *ε4* carriers, both in CSF and PET [2–4]. Multiple neuropathological studies have also shown higher neurofibrillary tangles in *ε4* carriers [6, 12, 51, 53, 57–60]. However, biomarker studies of *APOE-ε4* carriership effects on tau in CSF or PET are rather scarce and presented disparate results. Thus, some studies showed an association between the *ε4* allele and higher tau levels [21, 61], but others presented opposite associations [35] or no associations at all [8, 62]. These disparities may be related to disease stage of participants, with *ε4* carriers having higher tau burden in early clinical stages (i.e., CU or MCI) but not in more advanced stages, as has also been shown for Aβ burden [33, 63]. Together, our study is largely in line with established neuropathological results and adds more certainty to the inconclusive in vivo literature on the association of the *APOE-ε4* allele with tau pathology. Moreover, in the longitudinal analysis, we also found a trend-level association between the *ε4* allele and higher tau accumulation over time in neocortical Braak V/VI regions indicative of advanced tau pathology. The scarce previous studies examining *APOE-ε4* effects on longitudinal tau change also showed no or only limited associations between *APOE-ε4* carriership and tau accumulation. A previous study showed a higher rate of CSF tau accumulation over time in *ε4* carriers, but only in participants who were Aβ positive [8]. On the other hand, a previous longitudinal tau PET study did not find any significant *APOE-ε4* effect on regional tau accumulation rates in CU individuals from the Berkeley aging cohort, although this negative finding could be related to the relatively low number of *ε4* carriers in that study [64]. Finally, a recent longitudinal tau PET study found no *APOE-ε4* effect on tau accumulation rates among CU individuals after controlling for baseline Aβ load [65]. However, the study reported a marginally significant independent *APOE-ε4* effect on higher tau accumulation rates in a group of cognitively impaired individuals, but the interpretation of this finding is complicated by the mixed clinical composition of this group that included patients with MCI as well as typical and atypical AD dementia phenotypes.

Regarding non-PET measures, hippocampal volume and most of the cognitive measures were not associated with the *ε4* allele in our sample, although we observed a slightly lower memory performance in *ε4* carriers. Although in earlier stages, this is in line with the results found in a recent meta-analysis including only AD patients showing that *ε4* carriers present worse cognition than non-carriers only in the memory domain [66]. A review studying the effects of *APOE* on cognition also suggested that some of the cognitive deficits seen in *ε4* carriers might be related to AD pathology [67]. Therefore, the lack of an association between *APOE* and non-amnestic cognitive domain scores in our sample may stem from selecting only participants without dementia, as more robust *APOE* effects on neurodegeneration and cognition have been reported in participants with dementia [34, 68, 69].

Some previous neuropathological studies have raised the question whether *APOE-ε4* directly impacts tau pathology or only acts on tau through its effect on Aβ [10, 11]. There are two results in our study that would support the latter hypothesis. First, we observed a significant interaction between the *ε4* allele and Aβ status, where significant *APOE-ε4* effects on tau levels were only observed in the Aβ-positive group. Second, we found that Aβ levels significantly mediated the association between the *ε4* allele and tau pathology in all assessed brain regions. This reinforces the notion that the association between *ε4* carriership and tau burden is mainly driven by the pronounced *ε4* effect on increased Aβ pathology. However, another interesting outcome of our cross-sectional mediation analysis was that the mediating effect of Aβ was not complete for ERC tau levels, where the *ε4* allele also retained a significant Aβ-independent association with increased tau burden. This effect was also observed when limiting the analyses to Aβ-positive subjects only. These results are in agreement with a recent PET study, in which Aβ-independent associations of the *ε4* allele with tau levels were only found in the medial temporal lobe [9]. In the analog analysis of longitudinal tau PET data, we only found a trend towards higher tau accumulation in Braak V/VI regions in *ε4* carriers, but this effect was also fully mediated by baseline Aβ load. This mediation effect also remained significant when additionally adjusting the model for baseline tau load. Taken together, our results are in line with the hypothesis that the *APOE-ε4* allele might have an Aβ-independent effect on tau accumulation in the medial temporal cortex that occurs with aging [11], but that Aβ pathology is needed to accelerate tau pathology and facilitate its spread into the neocortex [11, 70–72]. However, it remains to be clarified whether this Aβ-independent *APOE-ε4* effect on ERC tau burden may also be associated with detrimental effects on cognition, as suggested by a recent study [73].

Previous studies examining sex*APOE-ε4 interactions on tau burden have led to some contradictory results, especially when comparing CSF biomarker and neuropathological studies. Thus, the large majority of studies measuring tau in CSF have provided evidence in favor of this interaction [21, 30, 31, 74], although not all [75]. One recent study could also replicate this finding using tau PET in MCI patients [74]. Neuropathological studies, on the other hand, did not find significant sex*APOE-ε4 interactions on tau pathology [30, 76]. A recent large-scale CSF study proposed that this interaction may only be present in the early phases of the disease (i.e., subjective cognitive decline and MCI) but decreases in later stages (Alzheimer’s dementia), which may explain the lack of results in neuropathological data [77]. Our present results would be in line with this notion as we only found a significant interaction in regions of early tau accumulation (i.e., the ERC), and sex-related differences in the *APOE-ε4* effect on tau burden were much lower for later regions (Table s2). Of note, in our Aβ mediation analysis stratified by sex, we found that only women presented the Aβ-independent effect of the *APOE-ε4* allele on tau levels in the ERC (Table s3). This suggests that the female-specific elevations in tau levels may stem from Aβ-independent pathways (e.g., sex hormones [78]). However, further investigation is needed to better understand these differences. Unfortunately, we were not able to perform meaningful longitudinal analysis stratified by sex due to the limited number of subjects in this subsample.

Among the main strengths of this study is that we investigated associations of the *ε2* allele with tau levels measured using PET, which allowed us to assess cross-sectional and longitudinal differences in regional tau deposition as opposed to the global results of CSF and cross-sectional results in neuropathological studies. There are also several limitations. First, although the sample size was large for a multitracer Aβ and tau PET imaging study, the final number of *ε2* carriers in this study was still limited, especially in the longitudinal analysis, which may have reduced the statistical power to detect more subtle associations with tau accumulation. However, at least in the cross-sectional analysis the effect size estimates were not indicative of even subtle differences in regional tau burden between *ε2* carriers and *ε3* homozygotes (Table 2). In this context, it is also important to note that statistical power was sufficient to fully replicate the previously reported *APOE-ε2* effect on reduced Aβ levels in this largely non-overlapping ADNI sample [19]. Another limitation is the use of linear models. We cannot discard that there might be some level of non-linearities in these associations, particularly in the cross-sectional analysis that covers a wide span of the clinical disease spectrum (CN to MCI). However, larger sample sizes would be needed to more comprehensively test these types of associations. A recent large-scale Aβ PET imaging study found that the Aβ load in *APOE-ε2ε4* participants was intermediate between that of *ε2* and *ε4* carriers, suggesting that the *ε2* allele may counteract some of the detrimental effects of the *ε4* allele [79]. Unfortunately, due to the low sample size of subjects with the *APOE-ε2ε4* genotype in our study (*n* = 7), we were not able to study these subjects as an independent group. The effects of this allele combination on tau PET imaging markers remain to be determined in larger samples with available tau PET acquisitions. Finally, in addition to structural MRI scans, the NIA-AA revised research criteria [80] also include [^18^F]fluorodeoxyglucose (FDG) PET as a neuroimaging marker of neurodegeneration. In the present study, we focused on MRI-derived hippocampal volumes instead of FDG PET measures due to the more limited availability of FDG PET and larger time lapse between this measure and Aβ and tau PET in the analyzed ADNI cohort. However, a previous study on multimodal neuroimaging correlates of the *APOE-ε2* allele indicated no significant differences in *APOE* effects on structural MRI and FDG PET-derived neurodegeneration measures [19].

In the context of this special issue on the Geneva roadmap for early diagnosis of AD based on biomarkers [32], our study contributes to phases 2 and 3 for both Aβ and tau PET imaging assessing the impact of genetics as covariate on biomarker results in healthy controls and in the early phases of disease. We would like to note that although at the time that the roadmap guidelines were formulated tau PET was still considered an emerging technology, this imaging biomarker has evolved enormously in recent years and is now shaping up to be considered a primary biomarker for diagnosis of AD [81].

## Conclusions

The *APOE-ε2* allele is associated with lower Aβ—but not tau—load, suggesting that lower Aβ deposition represents the main pathologic correlate of the decreased AD risk of *ε2* carriers. On the other hand, the elevated AD risk of *ε4* carriers may be related with higher tau load, although this also appears to be primarily mediated by the strong *APOE-ε4* effect on increased Aβ pathology, except in the ERC where Aβ-independent effects were significant.

## Supplementary information

ESM 1 (DOCX 461 kb)

## Data Availability

Data used in preparation of this article were obtained from the ADNI database (adni.loni.usc.edu).

## References

[1] Liu CC, Kanekiyo T, Xu H, Bu G (2013). Apolipoprotein e and Alzheimer disease: risk, mechanisms and therapy. Nat Rev Neurol..

[2] Jansen WJ, Ossenkoppele R, Knol DL, Tijms BM, Scheltens P, Verhey FRJ (2015). Prevalence of cerebral amyloid pathology in persons without dementia: a meta-analysis. JAMA J Am Med Assoc..

[3] Reiman EM, Chen K, Liu X, Bandy D, Yu M, Lee W (2009). Fibrillar amyloid-beta burden in cognitively normal people at 3 levels of genetic risk for Alzheimer’s disease. Proc Natl Acad Sci U S A..

[4] Jack CR, Wiste HJ, Weigand SD, Knopman DS, Vemuri P, Mielke MM (2015). Age, sex, and APOE ϵ4 effects on memory, brain structure, and β-Amyloid across the adult life Span. JAMA Neurol..

[5] Oyama F, Shimada H, Oyama R, Ihara Y (1995). Apolipoprotein E genotype, Alzheimer’s pathologies and related gene expression in the aged population. Mol Brain Res..

[6] Nagy ZS, Esiri MM, Jobst KA, Johnston C, Litchfield S, Sim E (1995). Influence of the apolipoprotein E genotype on amyloid deposition and neurofibrillary tangle formation in Alzheimer’s disease. Neuroscience..

[7] Slot RER, Verfaillie SCJ, Overbeek JM, Timmers T, Wesselman LMP, Teunissen CE (2018). Subjective Cognitive Impairment Cohort (SCIENCe): study design and first results. Alzheimer’s Res Ther.

[8] Buckley RF, Mormino EC, Chhatwal J, Schultz AP, Rabin JS, Rentz DM (2019). Associations between baseline amyloid, sex, and APOE on subsequent tau accumulation in cerebrospinal fluid. Neurobiol Aging..

[9] Therriault J, Benedet AL, Pascoal TA, Mathotaarachchi S, Chamoun M, Savard M (2020). Association of apolipoprotein e ϵ4 with medial temporal tau independent of amyloid-β. JAMA Neurol..

[10] Serrano-Pozo A, Qian J, Monsell SE, Betensky RA, Hyman BT (2015). APOEε2 is associated with milder clinical and pathological Alzheimer disease. Ann Neurol..

[11] Mungas D, Tractenberg R, Schneider JA, Crane PK, Bennett DA (2014). A 2-process model for neuropathology of Alzheimer’s disease. Neurobiol Aging. Elsevier Inc.

[12] Farfel JM, Yu L, De Jager PL, Schneider JA, Bennett DA (2016). Association of APOE with tau-tangle pathology with and without β-amyloid. Neurobiol Aging. Elsevier Inc.

[13] Shi Y, Yamada K, Liddelow SA, Smith ST, Zhao L, Luo W (2017). ApoE4 markedly exacerbates tau-mediated neurodegeneration in a mouse model of tauopathy. Nature. Nature Publishing Group.

[14] van der Kant R, Goldstein LSB, Ossenkoppele R (2020). Amyloid-β-independent regulators of tau pathology in Alzheimer disease. Nat Rev Neurosci. Springer US.

[15] Yamazaki Y, Zhao N, Caulfield TR, Liu CC, Bu G (2019). Apolipoprotein E and Alzheimer disease: pathobiology and targeting strategies. Nat Rev Neurol. Springer US.

[16] Reiman EM, Arboleda-Velasquez JF, Quiroz YT, Huentelman MJ, Beach TG, Caselli RJ (2020). Exceptionally low likelihood of Alzheimer’s dementia in APOE2 homozygotes from a 5,000-person neuropathological study. Nat Commun..

[17] Suri S, Heise V, Trachtenberg AJ, Mackay CE (2013). The forgotten APOE allele: a review of the evidence and suggested mechanisms for the protective effect of APOE e2. Neurosci Biobehav Rev. Elsevier Ltd.

[18] Corder EH, Saunders AM, Risch NJ, Strittmatter WJ, Schmechel DE, Gaskell PC (1994). Protective effect of apolipoprotein E type 2 allele for late onset Alzheimer disease. Nat Genet..

[19] Grothe MJ, Villeneuve S, Dyrba M, Bartrés-Faz D, Wirth M (2017). Multimodal characterization of older APOE2 carriers reveals selective reduction of amyloid load. Neurology..

[20] Morris JC, Roe CM, Xiong C, Fagan AM, Goate AM, Holtzman DM (2010). APOE predicts amyloid-beta but not tau Alzheimer pathology in cognitively normal aging. Ann Neurol..

[21] Altmann A, Tian L, Henderson VW, Greicius MD (2014). Sex modifies the APOE-related risk of developing Alzheimer disease. Ann Neurol..

[22] Toledo JB, Zetterberg H, Van Harten AC, Glodzik L, Martinez-Lage P, Bocchio-Chiavetto L (2015). Alzheimer’s disease cerebrospinal fluid biomarker in cognitively normal subjects. Brain..

[23] Chiang GC, Insel PS, Tosun D, Schuff N, Truran-Sacrey D, Raptentsetsang ST (2010). Hippocampal atrophy rates and CSF biomarkers in elderly APOE2 normal subjects. Neurology..

[24] Mattsson N, Schöll M, Strandberg O, Smith R, Palmqvist S, Insel PS (2017). 18 F-AV-1451 and CSF T-tau and P-tau as biomarkers in Alzheimer’s disease. EMBO Mol Med..

[25] Gordon BA, Friedrichsen K, Brier M, Blazey T, Su Y, Christensen J (2016). The relationship between cerebrospinal fluid markers of Alzheimer pathology and positron emission tomography tau imaging. Brain..

[26] Chhatwal JP, Schultz AP, Marshall G, Boot B, Gomez-Isla T, Dumurgier J (2016). Temporal T807 binding correlates with CSF tau and phospho-tau in normal elderly. Neurology..

[27] Leuzy A, Cicognola C, Chiotis K, Saint-Aubert L, Lemoine L, Andreasen N (2019). Longitudinal tau and metabolic PET imaging in relation to novel CSF tau measures in Alzheimer’s disease. Eur J Nucl Med Mol Imaging.

[28] Wolters EE, Ossenkoppele R, Verfaillie SCJ, Coomans EM, Timmers T, Visser D, et al. Regional [18F]flortaucipir PET is more closely associated with disease severity than CSF p-tau in Alzheimer’s disease. Eur J Nucl Med Mol Imaging. 2020:2866–78. 10.1007/s00259-020-04758-2 PMC7567681 32291510

[29] Meyer PF, Pichet Binette A, Gonneaud J, Breitner JCS, Villeneuve S (2020). Characterization of Alzheimer disease biomarker discrepancies using cerebrospinal fluid phosphorylated tau and AV1451 positron emission tomography. JAMA Neurol..

[30] Hohman TJ, Dumitrescu L, Barnes LL, Thambisetty M, Beecham G, Kunkle B (2018). Sex-specific association of apolipoprotein e with cerebrospinal fluid levels of tau. JAMA Neurol..

[31] Damoiseaux JS, Seeley WW, Zhou J, Shirer WR, Coppola G, Karydas A (2012). Gender modulates the APOE ε4 effect in healthy older adults: convergent evidence from functional brain connectivity and spinal fluid tau levels. J Neurosci..

[32] Frisoni GB, Boccardi M, Barkhof F, Blennow K, Cappa S, Chiotis K (2017). Strategic roadmap for an early diagnosis of Alzheimer’s disease based on biomarkers. Lancet Neurol..

[33] Ossenkoppele R, Van der Flier W, Zwan M, Adriaanse S, Boellaard R, Windhorst A (2013). Differential impact of apolipoprotein E genotype on distributions of amyloid load and glucose metabolism in Alzheimer’s disease. Neurology..

[34] Groot C, Sudre CH, Barkhof F, Teunissen CE, van Berckel BNM, Seo SW (2018). Clinical phenotype, atrophy, and small vessel disease in APOEε2 carriers with Alzheimer disease. Neurology..

[35] Mattsson N, Ossenkoppele R, Smith R, Strandberg O, Ohlsson T, Jögi J (2018). Greater tau load and reduced cortical thickness in APOE ε4-negative Alzheimer’s disease: a cohort study. Alzheimer’s Res Ther.

[36] Maass A, Landau S, Horng A, Lockhart SN, Rabinovici GD, Jagust WJ (2017). Comparison of multiple tau-PET measures as biomarkers in aging and Alzheimer’s disease. Neuroimage. Elsevier.

[37] Schöll M, Lockhart SN, Schonhaut DR, O’Neil JP, Janabi M, Ossenkoppele R (2016). PET imaging of tau deposition in the aging human brain. Neuron..

[38] Wolters EE, Golla SSV, Timmers T, Ossenkoppele R, van der Weijden CWJ, Scheltens P (2018). A novel partial volume correction method for accurate quantification of [(18)F] flortaucipir in the hippocampus. EJNMMI Res..

[39] Vogel JW, Mattsson N, Iturria-Medina Y, Strandberg OT, Schöll M, Dansereau C (2019). Data-driven approaches for tau-PET imaging biomarkers in Alzheimer’s disease. Hum Brain Mapp..

[40] Klunk WE, Koeppe RA, Price JC, Benzinger TL, Devous MD, Jagust WJ (2015). The Centiloid project: standardizing quantitative amyloid plaque estimation by PET. Alzheimer’s Dement.

[41] Wolf D, Bocchetta M, Preboske GM, Boccardi M, Grothe MJ (2017). Reference standard space hippocampus labels according to the European Alzheimer’s Disease Consortium-Alzheimer’s Disease Neuroimaging Initiative harmonized protocol: utility in automated volumetry. Alzheimers Dement. United States.

[42] Crane PK, Carle A, Gibbons LE, Insel P, Mackin RS, Gross A (2012). Development and assessment of a composite score for memory in the Alzheimer’s Disease Neuroimaging Initiative (ADNI). Brain Imaging Behav..

[43] Gibbons LE, Carle AC, Mackin RS, Harvey D, Mukherjee S, Insel P (2012). A composite score for executive functioning, validated in Alzheimer’s Disease Neuroimaging Initiative (ADNI) participants with baseline mild cognitive impairment. Brain Imaging Behav..

[44] Choi S, Mukherjee S, Gibbons LE, Sanders E, Jones RN, Tommett D, et al. Development and validation of language and visuospatial composite scores in ADNI. Alzheimer’s Dement. . 10.1002/trc2.12072 PMC7718716 33313380

[45] Salvadó G, Molinuevo JL, Brugulat-Serrat A, Falcon C, Grau-Rivera O, Suárez-Calvet M (2019). Centiloid cut-off values for optimal agreement between PET and CSF core AD biomarkers. Alzheimer’s Res Ther.

[46] La Joie R, Ayakta N, Seeley WW, Borys E, Boxer AL, DeCarli C, et al. Multisite study of the relationships between antemortem [11C]PIB-PET Centiloid values and postmortem measures of Alzheimer’s disease neuropathology. Alzheimer’s Dement. 2018:1–12. 10.1016/j.jalz.2018.09.001 PMC6368897 30347188

[47] Armstrong RA (2014). When to use the Bonferroni correction. Ophthalmic Physiol Opt J Br Coll Ophthalmic Opt. England.

[48] Hayes AF, Little TD (2018). Introduction to mediation, moderation, and conditional process analysis: a regression-based approach.

[49] Arenaza-Urquijo EM, Vemuri P (2018). Resistance vs resilience to Alzheimer disease. Neurology..

[50] Mattsson-Carlgren N, Andersson E, Janelidze S, Ossenkoppele R, Insel P, Strandberg O, et al. Aβ deposition is associated with increases in soluble and phosphorylated tau that precede a positive Tau PET in Alzheimer’s disease. Sci Adv. 2020;6. 10.1126/sciadv.aaz2387 PMC7159908 32426454

[51] Tiraboschi P, Hansen LA, Masliah E, Alford M, Thal LJ, Corey-Bloom J (2004). Impact of APOE genotype on neuropathologic and neurochemical markers of Alzheimer disease. Neurology..

[52] Lippa CF, Smith TW, Saunders AM, Hulette C, Pulaski-Salo D, Roses AD (1997). Apolipoprotein E-ε2 and Alzheimer’s disease: genotype influences pathologic phenotype. Neurology..

[53] Nicoll JAR, Savva GM, Stewart J, Matthews FE, Brayne C, Ince P (2011). Association between APOE genotype, neuropathology and dementia in the older population of England and Wales. Neuropathol Appl Neurobiol..

[54] Morris CM, Benjamin R, Leake A, McArthur FK, Candy JM, Ince PG (1995). Effect of apolipoprotein E genotype on Alzheimer’s disease neuropathology in a cohort of elderly Norwegians. Neurosci Lett..

[55] Blair CK, Folsom AR, Knopman DS, Bray MS, Mosley TH, Boerwinkle E (2005). APOE genotype and cognitive decline in a middle-aged cohort. Neurology..

[56] Wilson RS, Bienias JL, Berry-Kravis E, Evans DA, Bennett DA (2002). The apolipoprotein E ε2 allele and decline in episodic memory. J Neurol Neurosurg Psychiatry..

[57] Beffert U, Poirier J (1996). Apolipoprotein E, plaques, tangles and cholinergic dysfunction in Alzheimer’s disease. Ann N Y Acad Sci..

[58] Yu L, Boyle PA, Leurgans S, Schneider JA, Bennett DA (2014). Disentangling the effects of age and APOE on neuropathology and late life cognitive decline. Neurobiol Aging. Elsevier Ltd.

[59] Bennett DA, De Jager PL, Leurgans SE, Schneider JA (2009). Neuropathologic intermediate phenotypes enhance association to Alzheimer susceptibility alleles. Neurology..

[60] Sabbagh MN, Malek-Ahmadi M, Dugger BN, Lee K, Sue LI, Serrano G, et al. The influence of Apolipoprotein E genotype on regional pathology in Alzheimer’s disease. BMC Neurol. 2013;13:–7. 10.1186/1471-2377-13-44 PMC3654892 23663404

[61] Ossenkoppele R, Schonhaut DR, Schöll M, Lockhart SN, Ayakta N, Baker SL (2016). Tau PET patterns mirror clinical and neuroanatomical variability in Alzheimer’s disease. Brain..

[62] Gispert JD, Monté GC, Suárez-Calvet M, Falcon C, Tucholka A, Rojas S (2017). The APOE ε4 genotype modulates CSF YKL-40 levels and their structural brain correlates in the continuum of Alzheimer’s disease but not those of sTREM2. Alzheimer’s Dement Diagnosis, Assess Dis Monit..

[63] Lehmann M, Ghosh PM, Madison C, Karydas A, Coppola G, O’Neil JP (2014). Greater medial temporal hypometabolism and lower cortical amyloid burden in ApoE4-positive AD patients. J Neurol Neurosurg Psychiatry..

[64] Harrison TM, La Joie R, Maass A, Baker SL, Swinnerton K, Fenton L (2019). Longitudinal tau accumulation and atrophy in aging and Alzheimer disease. Ann Neurol..

[65] Jack CRJ, Wiste HJ, Weigand SD, Therneau TM, Lowe VJ, Knopman DS, et al. Predicting future rates of tau accumulation on PET. Brain. 2020; 10.1093/brain/awaa248 PMC7586089 33094327

[66] Emrani S, Arain HA, DeMarshall C, Nuriel T (2020). APOE4 is associated with cognitive and pathological heterogeneity in patients with Alzheimer’s disease: a systematic review. Alzheimers Res Ther.

[67] O’Donoghue MC, Murphy SE, Zamboni G, Nobre AC, Mackay CE (2018). APOE genotype and cognition in healthy individuals at risk of Alzheimer’s disease: a review. Cortex. Elsevier Ltd.

[68] Belloy ME, Napolioni V, Greicius MD. A quarter century of APOE and Alzheimer’s disease: progress to date and the path forward. Neuron. 2019. p. 820–38. 10.1016/j.neuron.2019.01.056 PMC6407643 30844401

[69] Martins CAR, Oulhaj A, De Jager CA, Williams JH (2005). APOE alleles predict the rate of cognitive decline in Alzheimer disease: a nonlinear model. Neurology..

[70] Price JL, Morris JC (1999). Tangles and plaques in nondemented aging and “preclinical” alzheimer’s disease. Ann Neurol..

[71] Musiek ES, Holtzman DM (2012). Origins of Alzheimer’s disease: reconciling cerebrospinal fluid biomarker and neuropathology data regarding the temporal sequence of amyloid-beta and tau involvement. Curr Opin Neurol..

[72] Pontecorvo MJ, Devous MD, Navitsky M, Lu M, Salloway S, Schaerf FW (2017). Relationships between flortaucipir PET tau binding and amyloid burden, clinical diagnosis, age and cognition. Brain..

[73] Weigand AJ, Thomas K, Bangen K, Eglit GM, Delano-Wood L, Gilbert P, et al. APOE interacts with tau PET to influence memory independently of amyloid PET. Alzheimer’s Dement. 2020:1–9. 10.1002/alz.12173 32886451

[74] Liu M, Paranjpe MD, Zhou X, Duy PQ, Goyal MS, Benzinger TLS (2019). Sex modulates the ApoE ε4 effect on brain tau deposition measured by 18F-AV-1451 PET in individuals with mild cognitive impairment. Theranostics..

[75] Sampedro F, Vilaplana E, de Leon JM, Alcolea D, Pegueroles J, Montal V (2015). APOE-by-sex interactions on brain structure and metabolism in healthy elderly controls. Oncotarget..

[76] Oveisgharan S, Buchman AS, Yu L, Farfel J, Hachinski V, Gaiteri C (2018). APOE ϵ2ϵ4 genotype, incident AD and MCI, cognitive decline, and AD pathology in older adults. Neurology..

[77] Mofrad RB, Tijms BM, Scheltens P, Barkhof F, van der Flier WM, AM Sikkes S, et al. Sex differences in CSF biomarkers vary by Alzheimer’s disease stage and APOE ε4 genotype. Neurology. 2020;10.1212/WNL.0000000000010629. 10.1212/WNL.0000000000010629 32788242

[78] Sundermann E, Panizzon M, Chen X, Andrews M, Galasko D, Banks S. Sex differences in Alzheimer’s-related Tau biomarkers and a mediating effect of testosterone. Biol Sex Diff. 2020:1–10. 10.1186/s13293-020-00310-x PMC7304096 32560743

[79] Insel PS, Hansson O, Mattsson-Carlgren N (2020). Association between apolipoprotein E ε2 vs ε4, age, and β-amyloid in adults without cognitive impairment. JAMA Neurol.

[80] Jack CR, Bennett D, Blennow K, Carrillo MC, Dunn B, Haeberlein SB (2018). NIA-AA Research Framework: toward a biological definition of Alzheimer’s disease. Alzheimer’s Dement. Elsevier Inc..

[81] Boccardi M, Dodich A, Albanese A, Gayet-Agéron A, Festari C, Ashton Nicholas J, et al. The Strategic Biomarker Roadmap for the validation of Alzheimer’s diagnostic biomarkers: methodological update. Eur J Nucl Med Mol Imaging. 2020. 10.1007/s00259-020-05120-2 PMC8175304 33688996

