# Supplementary material for: Differential associations of APOE-ε2 and APOE-ε4 alleles with PET-measured amyloid-β and tau deposition in older individuals without dementia
Source: Eur J Nucl Med Mol Imaging. 2021 Feb 1;48(7):2212–24. doi: 10.1007/s00259-021-05192-8 (PMC8175302; doi:10.1007/s00259-021-05192-8)
Supplement: Supplementary file 1 — (DOCX 461 kb) [file 259_2021_5192_MOESM1_ESM.docx]

**Differential associations of *APOE-ε2* and *APOE-ε4* alleles with PET-measured amyloid-β and tau deposition in older individuals without dementia**

Gemma Salvadó*, Michel J. Grothe*, Colin Groot, Alexis Moscoso, Michael Schöll, Juan Domingo Gispert & Rik Ossenkoppele, for the Alzheimer's Disease Neuroimaging Initiative

* These authors contributed equally

|  | All  (n=156) | *ε2* C  (n=10) | *ε3ε3*  (n=76) | | *ε4* C  (n=70) | | p |
| --- | --- | --- | --- | --- | --- | --- | --- |
| **Demographics** | | | | | | | |
| Age, years | 74.4 (6.8) [59 - 91] | 76.0 (8.4) [61 - 90] | 75.5 (6.3) [61 - 90] | | 73.1 (7.1)  [59 - 91] | | 0.091 |
| Women, n(%) | 79 (50.6) | 6 (60.0) | 33 (43.4) | | 40 (57.1) | | 0.210 |
| Education, years | 16.6 (2.4) | 15.5 (3.0) | 16.9 (2.3) | | 16.4 (2.5) | | 0.185 |
| Diagnosis, CU n(%) | 110 (70.5) | 6 (60.0) | 55 (72.4) | | 49 (70.0) | | 0.717 |
| MMSE | 28.6 (1.7) | 29.5 (1.1) | 28.7 (1.2) | | 28.1 (2.2) | | 0.072 |
| Time, years mean[range] | 1.6 (0.7)  [0.8 - 4.0] | 1.7 (0.5)  [0.9 - 2.2] | 1.6 (0.7)  [1 - 4] | | 1.6 (0.7)  [1 - 4] | | 0.878 |
| **Tau PET measurements (SUVr)** | | | | | | | |
| ERC | 1.19 (0.17) | 1.16 (0.13) | | 1.15 (0.15) | | 1.24 (0.18) | **0.004** |
| ITC | 1.25 (0.16) | 1.23 (0.10) | | 1.24 (0.18) | | 1.27 (0.15) | 0.314 |
| Braak V/VI | 1.09 (0.10) | 1.09 (0.07) | | 1.08 (0.11) | | 1.09 (0.08) | 0.581 |
| **Aβ PET measurement** | | | | | | | |
| Centiloids | 42.4 (43.8) | 3.4 (23.0) | | 31.9 (40.4) | | 59.5 (42.7) | **<0.001** |
| Aβ positive, n (%) | 100 (64.1%) | 3 (30.0) | | 40 (52.6) | | 57 (81.4) | **<0.001** |

**Table s1** Demographics and imaging and cognitive information of the subsample with longitudinal tau PET. Numbers show mean (SD) unless otherwise specified. Aβ status was defined as positive (negative) if Aβ load was higher (lower) than 12 Centiloids.

Abbreviations: CU, cognitively unimpaired; MMSE, Mini-Mental State Examination; SUVR, standardized uptake value ratio; ERC, entorhinal cortex; ITC, inferior temporal cortex; Aβ, amyloid-β.

| **Demographics** | ***APOE-ε2* - Men**  **(n=24)** | ***APOE-ε2* - Women**  **(n=21)** | ***APOE-ε3ε3* - Men**  **(n=125)** | ***APOE- ε3ε3* - Women**  **(n=132)** | ***APOE-ε4* - Men**  **(n=70)** | ***APOE-ε4* - Women**  **(n=92)** |
| --- | --- | --- | --- | --- | --- | --- |
| Age, years | 74.5 (7.0) | 75.0 (6.7) | 76.8 (7.2) | 73.3 (7.9) | 74.7 (7.8) | 71.7 (6.6) |
| Education, years | 16.8 (2.7) | 15.8 (2.5) | 17.1 (2.4) | 16.4 (2.4) | 16.8 (2.6) | 16.1 (2.5) |
| Diagnosis, CU n(%) | 13 (54.2) | 16 (76.2) | 75 (59.5) | 110 (82.1) | 39 (55.7) | 64 (69.6) |
| MMSE | 28.5 (1.9) | 28.9 (1.7) | 28.1 (2.9) | 28.7 (2.4) | 27.9 (2.9) | 28.4 (1.8) |

**Table s2:** Demographics of the study sample stratified by *APOE* and sex. Numbers show mean (SD) unless otherwise specified.

Abbreviations: CU, cognitively unimpaired; MMSE, Mini-Mental State Examination.

| **Demographics** | ***APOE-ε2* -**  **Aβ negative**  **(n=30)** | ***APOE-ε2* -**  **Aβ positive**  **(n=15)** | ***APOE-ε3ε3* -**  **Aβ negative**  **(n=131)** | ***APOE- ε3ε3* -**  **Aβ positive**  **(n=126)** | ***APOE-ε4* -**  **Aβ negative**  **(n=41)** | ***APOE-ε4* -**  **Aβ positive**  **(n=119)** |
| --- | --- | --- | --- | --- | --- | --- |
| Age, years | 72.9 (5.7) | 78.3 (7.6) | 73.3 (7.0) | 76.9 (8.1) | 70.1 (7.4) | 73.9(7.0) |
| Education, years | 16.2 (2.6) | 16.6 (2.7) | 16.8 (2.4) | 16.7 (2.4) | 16.7 (2.5) | 16.3 (2.6) |
| Diagnosis, CU n(%) | 17 (56.7) | 12 (80.0) | 100 (76.3) | 84 (66.7) | 32 (78.0) | 70 (58.8) |
| MMSE | 28.5 (1.8) | 29.0 (1.7) | 29.0 (1.2) | 27.8 (3.4) | 29.0 (1.1) | 27.9 (2.6) |

**Table s3:** Demographics of the study sample stratified by *APOE* and Aβ status. Numbers show mean (SD) unless otherwise specified. Aβ status was defined as positive (negative) if Aβ load was higher (lower) than 12 Centiloids.

Abbreviations: CU, cognitively unimpaired; MMSE, Mini-Mental State Examination; Aβ, amyloid-β.

|  | ***APOE-ε2*** | | ***APOE-ε4*** | |
| --- | --- | --- | --- | --- |
|  | β [95%CI] | p_corr_ | β [95%CI] | p_corr_ |
| **Tau measurements** | | | | |
| ERC | 0.03 [-0.17, 0.39] | 0.940 | **0.51 [0.33, 0.70]** | **<0.001** |
| ITC | -0.10 [-0.26, 0.10] | 0.787 | **0.30 [0.08, 0.49]** | **0.004** |
| Braak V-VI | -0.12 [-0.32, 0.08] | 0.689 | **0.27 [0.03, 0.49]** | **0.012** |
| **Aβ measurement** | | | | |
| Centiloids | -0.31 [-0.45, -0.16] | 0.067 | **0.64 [0.42 - 0.82]** | **<0.001** |
| **MRI measurement** | | | | |
| Hippocampal volumes | -0.02 [-0.35, 0.26] | 0.980 | -0.08 [- 0.24, 0.10] | 0.624 |
| **Cognitive composite measures** | | | | |
| Episodic memory | -0.05 [-0.30, 0.24] | 0.932 | -0.17 [-0.33, 0.01] | 0.100 |
| Executive function | -0.04 [-0.27, 0.23] | 0.952 | -0.10 [-0.27, 0.07] | 0.431 |
| Language | 0.05 [-0.25, 0.32] | 0.926 | 0.03 [-0.14, 0.22] | 0.912 |
| Visuospatial functioning | 0.18 [-0.12, 0.48] | 0.431 | -0.03 [-0.22, 0.18] | 0.946 |

**Table s4:** Linear regression parameters (standardized β) for the association of *APOE-ε2* and *APOE-ε4* genotypes with tau PET, Aβ PET, MRI and cognition measurements. *APOE-ε3ε3* participants were selected as the reference group for all comparisons*.* The model included age, sex, education and diagnosis as covariates. Statistically significant results (p<0.05) are shown in bold. P-values were adjusted for multiple comparisons using Dunnet's test.

Abbreviations: Aβ, amyloid-β; MRI, magnetic resonance imaging; CU, cognitively unimpaired; CI, confidence.

|  | ***APOE-ε2* - Men**  **(n=24)** | | ***APOE-ε2* - Women**  **(n=21)** | | | ***APOE-ε4* - Men**  **(n=70)** | | ***APOE-ε4* - Women**  **(n=92)** | |
| --- | --- | --- | --- | --- | --- | --- | --- | --- | --- |
|  | β [95%CI] | p | | β [95%CI] | p | β [95%CI] | p | β [95%CI] | p |
| **Tau measurements** | | | | | | | | | |
| ERC | 0.16 [-0.20, 0.52] | 0.475 | | -0.08 [-0.43, 0.27] | 0.702 | **0.33 [0.08, 0.57]** | **0.028** | **0.63 [0.43 - 0.83]** | **<0.001** |
| ITC | -0.12 [-0.48, 0.25] | 0.600 | | -0.09 [-0.46, 0.27] | 0.678 | 0.24 [-0.00, 0.49] | 0.106 | **0.32 [0.10 - 0.53]** | **0.015** |
| Braak V/VI | -0.20 [-0.57, 0.17] | 0.381 | | -0.06 [-0.43 - 0.32] | 0.802 | 0.25 [-0.00, 0.50] | 0.101 | **0.26 [0.04 - 0.48]** | **0.050** |
| **Aβ measurement** | | | | | | | | | |
| Centiloids | -0.34 [-0.68, -0.01] | 0.091 | | -0.26 [-0.61, 0.09] | 0.225 | **0.57 [0.34 - 0.80]** | **<0.001** | **0.68 [0.48 - 0.89]** | **<0.001** |

**Table s5:** Linear regression parameters (standardized β) for the association between *APOE* and PET biomarkers stratified by sex groups. *APOE-ε3ε3* participants were selected as the reference group for all *APOE* comparisons. The model included age, education and diagnosis as covariates. Statistical significant results (p<0.05) are shown in bold.

Abbreviations: ERC, entorhinal cortex; ITC, inferior temporal cortex; Aβ, amyloid-β

|  | ***APOE-ε2* - Aβ negative**  **(n=30)** | | ***APOE-ε2* - Aβ positive**  **(n=15)** | | | ***APOE-ε4* - Aβ negative**  **(n=41)** | | ***APOE-ε4* - Aβ positive**  **(n=119)** | |
| --- | --- | --- | --- | --- | --- | --- | --- | --- | --- |
|  | β [95%CI] | p | | β [95%CI] | p | β [95%CI] | p | β [95%CI] | p |
| **Tau measurements** | | | | | | | | | |
| ERC | **0.48 [0.15 - 0.81]** | **0.017** | | -0.12 [-0.53, 0.29] | 0.619 | -0.02 [-0.31 - 0.27] | 0.916 | **0.50 [0.31 - 0.70]** | **<0.001** |
| ITC | 0.21 [-0.12, 0.54] | 0.296 | | -0.10 [-0.53, 0.33] | 0.709 | -0.11 [-0.41 - 0.18] | 0.530 | 0.23 [0.02 - 0.44] | 0.066 |
| Braak V/VI | 0.08 [-0.25, 0.41] | 0.685 | | -0.08 [-0.51, 0.36] | 0.774 | 0.01 [-0.28 - 0.30] | 0.954 | 0.18 [-0.03 - 0.39] | 0.152 |

**Table s6:** Linear regression parameters (standardized β) for the association between *APOE* and PET biomarkers stratified by Aβ status. *APOE-ε3ε3* participants were selected as the reference group for all *APOE* comparisons. The model included age, education and diagnosis as covariates. Statistical significant results (p<0.05) are shown in bold. Aβ status was defined as positive (negative) if Aβ load was higher (lower) than 12 Centiloids.

Abbreviations: ERC, entorhinal cortex; ITC, inferior temporal cortex; Aβ, amyloid-β.


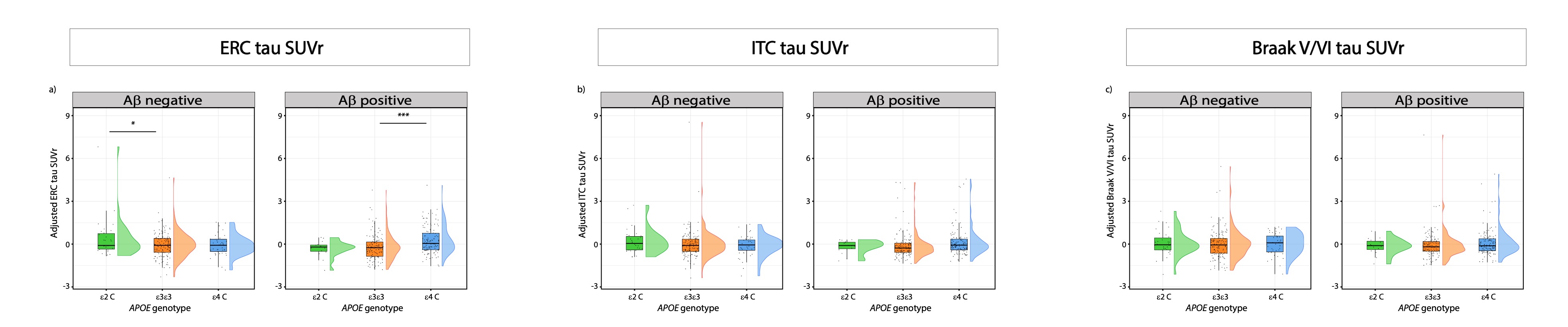


**Figure s1:** Associations of *APOE-ε2* and *APOE-ε4* alleles with cross-sectional measures and tau burden by Aβ status. Tau regions studied were: ERC (a), ITC (b), Braak V/VI (c). PET measures are adjusted by age, sex, education and diagnosis. Boxplots show median values (middle line) with lower and upper hinges corresponding to the first and third quartiles. Dots represent individual adjusted PET measures, with violin plots showing their distribution. Aβ status was defined as positive (negative) if Aβ load was higher (lower) than 12 Centiloids.

Abbreviations: Aβ, amyloid-β; ERC, entorhinal cortex; ITC, inferior temporal cortex; SUVR, standardized uptake value ratio.

* p<0.05; *** p<0.001

|  | ***APOE-ε2**Sex** | | ***APOE-ε4**Sex** | | ***APOE-ε2**Aβ status** | | ***APOE-ε4**Aβ status** | |
| --- | --- | --- | --- | --- | --- | --- | --- | --- |
|  | β [95%CI] | p_corr_ | β [95%CI] | p_corr_ | β [95%CI] | p_corr_ | β [95%CI] | p_corr_ |
| **Tau measurements** | | | | | | | | |
| ERC | -0.18  [-0.82, 0.24] | 0.749 | 0.39  [0.03 - 0.76] | 0.067 | -0.35  [-0.88, 0.07] | 0.462 | **0.62**  **[0.28, 0.91]** | **0.004** |
| ITC | 0.04  [-0.33, 0.40] | 0.993 | 0.12  [-0.26, 0.56] | 0.736 | -0.13  [-0.55, 0.20] | 0.878 | 0.38  [-0.03, 0.67] | 0.152 |
| Braak V/VI | 0.13  [-0.27, 0.59] | 0.886 | 0.12  [-0.29, 0.54] | 0.768 | -0.07  [-0.50, 0.39] | 0.966 | 0.30  [-0.13, 0.62] | 0.318 |
| **Aβ measurement** | | | | | | | | |
| Centiloids | 0.08  [-0.23, 0.39] | 0.951 | 0.15  [-0.27, 0.48] | 0.695 | - | - | - | - |

**Table s7:** Linear regression parameters (standardized β) for the interaction between *APOE* and sex, and *APOE* and Aβ status on tau and Aβ load. *APOE-ε3ε3* men and *APOE-ε3ε3* Aβ negative were selected as the reference group for sex and Aβ status comparisons, respectively*.* Aβ status was positive (negative) if Aβ load was higher (lower) than 12 Centiloids [45,46]. All models included age, education and diagnosis as covariates. Statistically significant results (p<0.05) are shown in bold. P-values were adjusted for multiple comparisons using a multivariate *t* distribution.

|  | **Men** | | | | | | **Women** | | | | |
| --- | --- | --- | --- | --- | --- | --- | --- | --- | --- | --- | --- |
|  | Total effect,  (c) | Mediated effect,  (a_1_·b_1_) | Direct effect,  (c') | Percentage mediation  (a_1_·b_1_/c) | Percentage direct  (c'/c) | Total effect, (c) | | Mediated effect,  (a_1_·b_1_) | Direct effect,  (c') | Percentage mediation  (a_1_·b_1_/c) | Percentage direct  (c'/c) |
| ERC | 0.063 (0.028) | 0.037 (0.014) | n.s. | 59% | n.s. | 0.130 (0.027) | | 0.081 (0.020) | 0.050 (0.023) | 62% | 38% |
| ITC | n.s. | n.s. | n.s. | n.s. | n.s. | 0.077 (0.034) | | 0.092 (0.026) | n.s. | 100% | n.s. |
| Braak V/VI | n.s. | n.s. | n.s. | n.s. | n.s. | *0.037 (0.020)* | | 0.048 (0.015) | n.s. | 100% | n.s. |

**Table s8:** Parameters of the cross-sectional mediation analyses stratified by sex. The dependent variable (X) was the *APOE-ε4* allele, the mediator (M) was Aβ burden, measured as Centiloids, and the dependent variable (Y) was tau burden, measured as SUVR, in the different ROIs. The first three columns of each analysis show path weights (SE), while the last two show the percentage over the total effect. Only paths that were statistically significant (p<0.05) or showed a trend to significance (p<0.1, in italics) are shown. All models were adjusted by age, education and diagnosis.

Abbreviations: ERC, entorhinal cortex; ITC, inferior temporal cortex; n.s., not significant path; Aβ, amyloid-β; ROI, region of interest; SE, standard error; SUVR, standardized uptake value ratio.

|  | **Aβ negative** | | | | | | **Aβ positive** | | | | |
| --- | --- | --- | --- | --- | --- | --- | --- | --- | --- | --- | --- |
|  | Total effect,  (c) | Mediated effect,  (a_1_·b_1_) | Direct effect,  (c') | Percentage mediation  (a_1_·b_1_/c) | Percentage direct  (c'/c) | Total effect, (c) | | Mediated effect,  (a_1_·b_1_) | Direct effect,  (c') | Percentage mediation  (a_1_·b_1_/c) | Percentage direct  (c'/c) |
| ERC | n.s. | n.s. | n.s. | n.s. | n.s. | 0.110 (0.028) | | 0.047 (0.016) | 0.063 (0.025) | 43% | 57% |
| ITC | n.s. | n.s. | n.s. | n.s. | n.s. | *0.069*  *(0.038)* | | 0.058  (0.020) | n.s. | 84% | n.s. |

**Table s9:** Parameters of the cross-sectional mediation analyses stratified by Aβ status. The dependent variable (X) was the *APOE-ε4* allele, the mediator (M) was Aβ burden, measured as Centiloids, and the dependent variable (Y) was tau burden, measured as SUVR, in the different ROIs. The first three columns of each analysis show path weights (SE), while the last two show the percentage over the total effect. Only paths that were statistically significant (p<0.05) or showed a trend to significance (p<0.1, in italics) are shown. All models were adjusted by age, education and diagnosis. Aβ status was defined as positive (negative) if Aβ load was higher (lower) than 12 Centiloids.

Abbreviations: ERC, entorhinal cortex; ITC, inferior temporal cortex; n.s., not significant path; Aβ, amyloid-β; ROI, region of interest; SE, standard error; SUVR, standardized uptake value ratio.

**Figure s2: Mediation analysis limited to the Aβ positive group.** Mediation effect of Aβ on the association of *APOE-ε4* with tau burden in the ERC (a) and ITC (b). Dark green lines show the total effect of *APOE-ε4* allele on tau burden, light green lines show the direct effect (*i.e.* without mediation), and blue lines depict the Aβ mediation effect. Path-weights are only shown for significant paths and are displayed as (unstandardized) beta values with standard errors in brackets. Significance of the indirect effect was determined using bootstrapping with 5,000 iterations. All models were adjusted by age, sex, education and diagnosis. Aβ status was defined as positive if Aβ load was higher than 12 Centiloids.

Abbreviations: Aβ, amyloid-β; ERC, entorhinal cortex; ITC, inferior temporal cortex.

|  | ***APOE-ε2**time** | | ***APOE-ε4**time** | |
| --- | --- | --- | --- | --- |
|  | β [95%CI] | p_corr_ | β [95%CI] | p_corr_ |
| ERC | -0.10 [-0.25, 0.07] | 0.387 | 0.08 [-0.04, 0.15] | 0.210 |
| ITC | -0.07 [-0.17, 0.07] | 0.593 | 0.08 [-0.01, 0.15] | 0.118 |
| Braak V/VI | -0.01 [-0.18, 0.18] | 0.999 | 0.10 [-0.02 - 0.18] | 0.057 |

**Table s10:** Linear mixed model regression parameters (standardized β) for the association of *APOE-ε2* and *APOE-ε4* genotypes with longitudinal rates of regional tau SUVR change over time. *APOE-ε3ε3* participants were selected as the reference group for all comparisons. The model included age at baseline, sex, education and diagnosis as covariates. P-values were adjusted for multiple comparisons using a multivariate *t* distribution.

Abbreviations: Aβ, amyloid-β; ERC, entorhinal cortex; ITC, inferior temporal cortex; CI, confidence intervals.
